# A systematic review of factors influencing participation in two types of malaria prevention intervention in Southeast Asia

**DOI:** 10.1186/s12936-021-03733-y

**Published:** 2021-04-20

**Authors:** Breagh Cheng, Saw Nay Htoo, Naw Pue Pue Mhote, Colleen M. Davison

**Affiliations:** 1 Department of Public Health Sciences, Queen’s University, 62 Fifth Field Company Lane, Kingston, ON K7L3N6 Canada; 2 Burma Medical Association, Mae Sot, Thailand; 3 Health Information Systems Working Group, Mae Sot, Thailand

**Keywords:** Malaria, Insecticide-treated nets, Systematic review, Southeast Asia

## Abstract

**Background:**

Multi-pronged malaria elimination strategies are increasingly being considered for accelerating efforts against malaria transmission in Southeast Asia. Two malaria prevention interventions used in in the region are insecticide-treated bed-nets (ITNs) and mass drug administration (MDA). Universal access to ITNs is recommended and high population coverage (e.g. above 80%) is needed during MDA initiatives to maximize the impact of these interventions. However, variability in ITN use and individual MDA participation exists. This systematic review aims to provide a summary and overview of literature discussing factors influencing uptake of these two malaria control strategies in Southeast Asian countries.

**Methods:**

A search of OVID Embase, OVID MEDLINE, Cochrane Central Register of Controlled Trials, Web of Science, OpenGrey, ProQuest, and Google Scholar was undertaken in February 2020. English-language publications with any study design using data from any of the ten member countries of the Association of Southeast Asian Nations were eligible for inclusion. In addition, reference lists of identified articles were manually searched. Websites for relevant international agencies were also searched to identify related grey literature.

**Results:**

The review identified thirty publications that met the inclusion and exclusion criteria. Most discussed ITN use (n = 18) and were relevant to populations in Myanmar (n = 14). All MDA studies were published after 2016, whereas included ITN studies spanned from 1998 to 2020. Seven main themes emerged across the studies. Knowledge of malaria and attitudes towards ITNs were emphasized as key factors associated with ITN use. For MDA participation, key factors included the importance of positive attitudes towards the program, the influence of indirect costs and incentives, and the tendency for group decision-making.

**Conclusions:**

As countries in Southeast Asia continue to work towards becoming malaria-free by 2030, the knowledge and attitudes of local population sub-groups should be assessed and incorporated into the planning and implementation of malaria prevention activities. The role of incentives and group decision making should also be considered particularly as they relate to MDA. There is need for ongoing involvement of health educators, the continuation of implementation research and the prioritization of community engagement efforts alongside malaria interventions in the region.

**Supplementary Information:**

The online version contains supplementary material available at 10.1186/s12936-021-03733-y.

## Background

Marked reductions in malaria incidence have been made in Southeast Asia over the past two decades. Southeast Asian countries, such as Singapore and Brunei, have maintained malaria-free status, the remaining countries have committed to the goal of eliminating malaria by the year 2030 [1]. Despite achieving notable decreases in morbidity and mortality, malaria remains an important disease burden in the region. Nearly 8 million combined cases of malaria were reported across Brunei, Cambodia, Indonesia, Laos, Malaysia, Myanmar, the Philippines, Singapore, Thailand and Vietnam, with over 200 million people in this region at risk of contracting malaria in 2018 [2, 3]. Aside from direct health consequences, malaria diverts time away from income-generating activities leading to reduced household income, which can be especially severe for poor households, highlighting additional economic and social implications for the region [4, 5]. Malaria has also been reported to adversely impact the educational attainment of school children, with decreased school performance as the number of malaria infections increase [6].

Insecticide-treated bed nets (ITNs) or long-lasting insecticidal nets (LLIN) have been a cornerstone of malaria control for decades [7]. These interventions have been shown to be generally effective for malaria prevention, although their impact on malaria transmission in Southeast Asia is lower compared to in sub-Saharan Africa due in part to differences in the outdoor biting and outdoor resting behaviour of relevant vectors of mosquitoes [8, 9]. To accelerate malaria elimination efforts in Southeast Asia, mass drug administration (MDA) has increasingly been considered as part of multi-pronged strategies [10]. These interventions have been co-implemented in several Southeast Asian countries including as Cambodia, Vietnam, and Laos, and have been shown to help decrease malaria incidence [11]. The impact of MDA relies on high individual uptake of MDA in the target population; given that malaria transmission intensity partly depends on vectorial capacity, the MDA coverage required is likely higher than 80% of uptake to interrupt malaria transmission [12–14]. Modelling studies predict that coupling of ITNs and MDA drastically improves the likelihood of elimination in countries in the Greater Mekong Sub-Region. There is a possibility that malaria will be eliminated by the year 2025 in Cambodia, Indonesia, Laos, and Myanmar with the adoption of these combined approaches or scale up of these interventions for countries such as Bhutan and Thailand [15, 16]. Understanding factors affecting intervention uptake in previous and current intervention programmes is necessary to understand and address potential barriers to effective and equitable implementation and scale-up [17]. The purpose of this systematic review was to provide an overview of factors that contribute to the use of ITNs and to individual participation in MDA programmes in Southeast Asia. Specifically, this review aimed to quantify the current state of research aimed at understanding uptake of these malaria interventions, describe any patterns in uptake and identify key gaps in knowledge needed to develop effective elimination strategies for malaria in Southeast Asia [18].

## Methods

The methodology for this systematic review followed the procedure proposed by Arskey and O’Malley [19]. These steps include developing the research question, searching for relevant literature, selecting publications, charting data, and collating, summarizing and reporting results. Reporting of this review followed the Preferred Reporting of Items for Systematic Reviews and Meta-Analyses (PRISMA) guidelines. The review was guided by the research question: What has been described about the determinants affecting the use of ITNs and/or individual participation in MDA in Southeast Asian countries?

### Search strategy

To search for and identify relevant studies, a search strategy was developed in consultation with an experienced health sciences research librarian. A systematic search was conducted on February 21, 2020 in seven academic and grey literature databases: OVID Embase; OVID MEDLINE; Cochrane Central Register of Controlled Trials (CENTRAL); Web of Science; OpenGrey; ProQuest; and Google Scholar. Additionally, a manual search was also conducted of reference lists of included publications and websites belonging to the Shoklo Malaria Research Unit and international organizations including: the World Health Organization (WHO) Index Medicus for the South-East Asia Region, Roll Back Malaria Partnership, International Organization for Migration, United Nations High Commission for Refugees, United Nations Children’s Fund, The United States Agency for International Development, President’s Malaria Initiative, Partners for Development, Asia Pacific Leaders Malaria Alliance, Malaria No More, and Population Services International [20].

Key search terms included: “malaria, “bed net,” “mass drug administration,” and “Southeast Asia” as well as the names of the ten ASEAN member countries [21]. Given that Web of Science does not have Subject Headings, only keywords in the search strategy were searched, including mass drug administration or antimalaria* or anti-malaria*. A complete list of search terms used can be found in the Additional file 1.

### Eligibility criteria

Relevant publications were selected based on the inclusion and exclusion criteria that were defined a priori (Box 1). Given this study’s aim to provide an overview of available literature on factors affecting ITN use and/or individual MDA participation, no restrictions were placed on published year or study design. Publications were included if they explicitly had as part of their objectives to assess one or more determinants of delivery affecting either the use of ITNs and/or individual-level MDA participation for malaria prevention in a Southeast Asian country belonging to the Association of Southeast Asian Nations (ASEAN). Given that English is the primary working language of the lead author, eligible publications were restricted to the English language. Publications were excluded if they were not focused on malaria, did not discuss ITNs, LLINs or MDA or collected data from any countries not belonging to the ASEAN group.

**Box 1:** Inclusion and exclusion criteria

Criterion	Inclusion	Exclusion
Language	English	Non-English studies
Study intervention focus	ITNs, LLINs, and MDA	Other vector control measures (ex. Mosquito coils, insecticide-treated hammocks, topical repellents, insecticide-treated clothing)
Data collection setting	At least one of or any combination of the ten countries belonging to ASEAN, including Brunei, Cambodia, Indonesia, Laos, Malaysia, Myanmar, the Philippines, Singapore, Thailand and Vietnam	Any country not belonging to the ASEAN group
Literature focus	Has explicitly stated objectives to identify one or more determinants of ITN use and/or participation in MDA	Does not explicitly state objectives or made an insubstantial reference to factors influencing ITN use and/or participation in MDA; Full text unobtainable

### Study selection

The final search results were imported into the online review manager Covidence (version 2020) and duplicate articles were removed. Abstract and full-text screening were independently reviewed by two investigators (BC and a graduate student) and excluded publications that did not meet the inclusion criteria. Any discordances were resolved through discussion and reaching consensus between the lead author and the graduate student volunteer.

### Synthesis of results

The fourth step was charting selected articles. Publications meeting inclusion criteria were grouped based on the intervention of focus and then summarized by key characteristics, including first author and year of publication, setting of data collection, sample characteristics, and study design. An inductive approach was used to assess the extracted data for common themes related to the barriers and facilitators for ITN use and/or MDA participation.

## Results

**Fig. 1:**
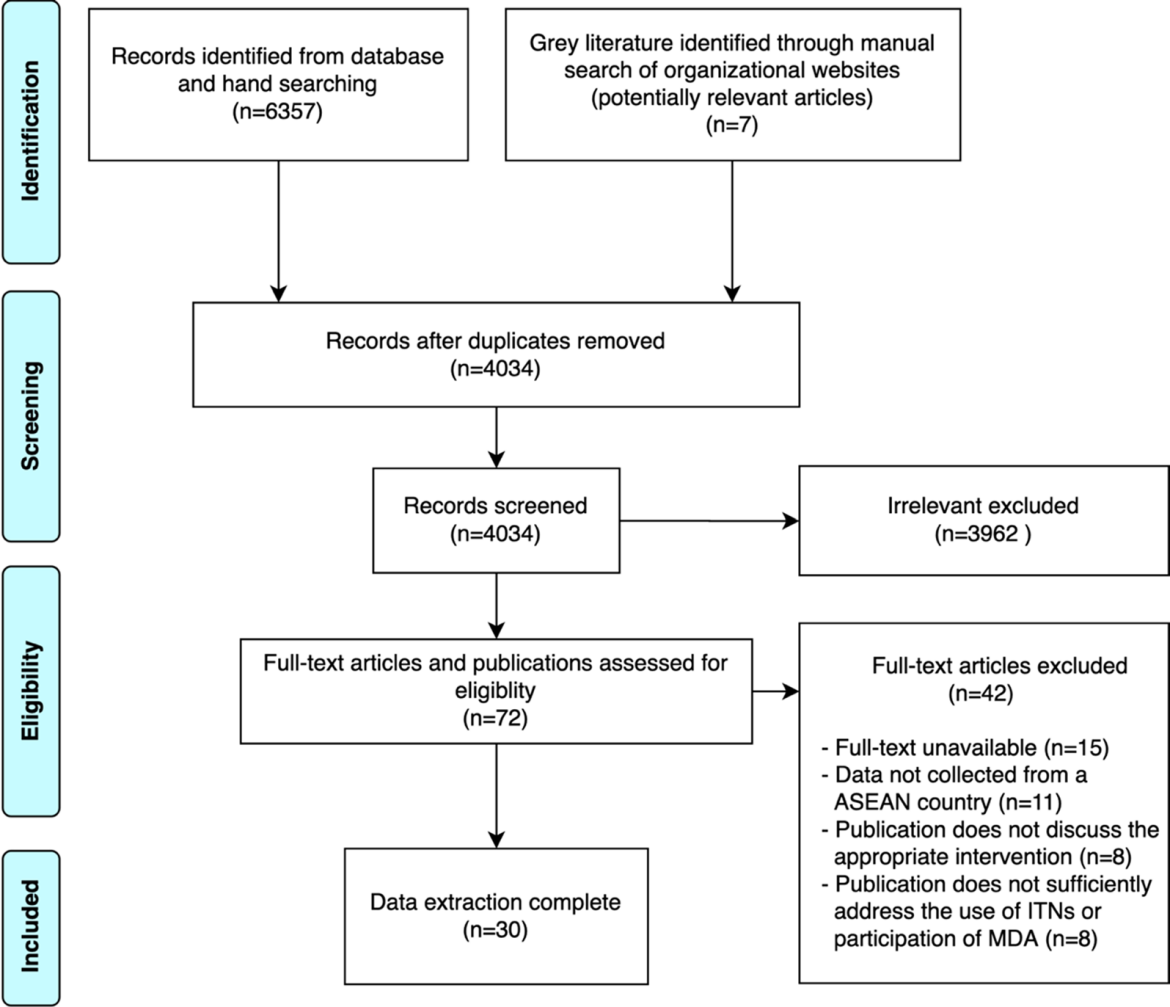
PRISMA flow diagram outlining the search and study selection process

A total of 6364 titles and abstracts were screened. After assessing abstracts and full-text articles based on inclusion and exclusion criteria, thirty eligible publications consisting of academic and grey literature were included. The process of article selection followed PRISMA guidelines [22] (see Fig. 1 for PRISMA flow chart). Summaries of the main characteristics of the included papers are presented in Additional files 2 and 3.

Of the included publications, eighteen (60%) discussed ITN use, eleven discussed MDA, and one study discussed both ITN and MDA. All MDA studies were published within the last 5 years (2016–2020) whereas only a third of included ITN studies were published in the same period and the rest spanned between 1998–2015. Of the eight quantitative cross-sectional studies, most (n = 5) focused on ITN use. Half of the sixteen (53%) publications that followed qualitative or mixed methodology research designs focused on ITN use. One systematic review fulfilled the inclusion criteria and five publications were grey literature documents (17%), of which four discussed ITN use. Of the twenty-five studies with a single country focus, publications discussed intervention uptake in Myanmar (n = 14, of which 10 focused on ITN use), Cambodia (n = 5), Laos (n = 3), Vietnam (n = 1), Thailand (n = 1), and Malaysia (n = 1). Five publications had a multi-country focus. Publications examined factors influencing ITN use and/or MDA participation among the general population, as well as mobile and migrant groups. One study focused on factors specific to ITN use among the Laos military [1] and one study had a focus on factors affecting MDA participation as perceived by policymakers [23].

While factors describing intervention uptake fell broadly into a total of seven themes, it is recognized that ITNs and MDA are different types of interventions. ITNs have been in routine use for extended periods in Southeast Asia whereas MDA projects are typically conducted by academic institutions as research projects and have been more recently implemented. Thus, the factors found to affect uptake of these interventions have been summarized in separate additional files. Additional file 4 presents themes describing factors related to ITN use and Additional file 5 is specific to MDA participation.

### Themes describing factors related to ITN use

#### Access and delivery

Insufficient access to bed nets was a key factor associated with delayed possession and use of bed nets, with the most common reason being absence during net distribution [24–26]. Other reasons associated with non-ITN use include insufficient household net supply and selective distribution practices by staff involved in net distribution among general and specific populations [25–28].

#### Costs and benefits

ITNs costs and benefits were both related to their use. The cost of ITNs was a common reason reported for not owning or using nets, or not replacing older or damaged nets [25, 28–33]. Individuals were motivated to use ITNs by perceptions of bed nets as beneficial [32] due to reasons such as, preventing malaria infection [34] and sense of privacy [28, 33].

#### Malaria knowledge

Greater knowledge about malaria transmission was significantly associated with increased likelihood of ITN use in most analytical studies [32, 34–36] except for in one quantitative study that found a non-significant association between net use and knowledge of malaria aetiology in Thailand [32]. Poor awareness of malaria transmission was related to some families using nets for reasons other than malaria prevention, such as for fishing and for warmth [29, 37, 38].

#### Intervention knowledge

Awareness of ITNs and their role in malaria prevention was related to their increased use. Individuals who reported receiving information about impregnated bed nets from malaria workers had a higher likelihood of ITN use and conversely, not knowing about ITNs was a primary reason for their non-use [32].

#### Attitudes and perceptions

Using ITNs was related to a belief in their malaria risk and recognition of malaria as a serious health hazard [25, 38, 39]. Some subgroups, such as male youth in Cambodia and Vietnam, refrained from using nets due to self-perceived low vulnerability to malaria [28, 40], and instead prioritized nets for use by mothers and children under five [28]. ITNs were also not prioritized for use following participation in leisure activities, such as late-night TV-watching [28, 30, 33]. Low ITN use was reported among Laos soldiers, migrant workers and mobile male youth due to the inconvenience of carrying nets while on the move [26].

Attitudes regarding the comfort and perceived effectiveness of ITNs also seemed to be associated with the characteristics of the interventions themselves [25, 26, 28, 37–39, 41, 42]. For instance, ITN use was discontinued after experiencing excessive heat [25, 39, 41], unpleasant insecticide smells [26] or texture [37], as well as health concerns (e.g. burning sensations) while sleeping underneath the net [37]. Larger households preferred bigger nets with finely woven meshing, the larger size to accommodate more people and the fine meshing because of the perception that other meshing might allowed insects inside the net [28, 31, 37, 39, 43]. Perceived poor durability due to rapid deterioration after net washing also contributed to low net use [28].

#### Personal characteristics

ITN use was associated with several demographic characteristics. Wealthier households living in urban locations near health facilities were positively associated with ITN ownership [35, 44, 45]. The likelihood of living in a household with at least one ITN decreases as age of a household member increased [45]. A significantly higher likelihood of sleeping under an ITN was also associated with higher altitude regions, smaller household sizes, and if a household was headed by farmers and fisherman compared to household heads who were skilled workers [36]. Ethnic male youth in Cambodia and adult males were less likely to use ITNs compared to women and children due to behavioural risk patterns and the tendency for males to have temporary sleeping arrangements [28, 31, 33].

### Themes describing factors related to individual MDA participation

#### Access and delivery

Accessing remote communities targeted for MDA implementation was a time and resource-intensive endeavour that is linked to frequent programme staff turnover [41]. Further challenges in obtaining policymaker support and government approval prior to MDA study initiation has contributed to implementation delays [23]. Absence during the MDA campaign due to work responsibilities was commonly cited as a reason for non-participation [24, 34].

#### Costs and incentives

Direct and indirect costs were related to participation in MDA. Non-participation stemmed from unwillingness to divert time away from work, concerns for the economic implications associated with contracting malaria and management of perceived side effects of taking anti-malarial drugs [25, 46]. While financial compensation was associated with a greater likelihood of participation, participation rates did not change significantly in its absence [41, 47]. Community members also strongly valued additional incentives associated with the intervention such as the provision of free essential medical services and responses to other community needs, such as installing water pumps, as well as attention to their health concerns at the same time as door-to-door MDA delivery [48–51]. These services were seen as a demonstration of genuine care for the community’s health and social concerns, which was associated with increased confidence in the intervention and ultimately played a motivating reason for participation [48].

#### Malaria knowledge

Knowledge of malaria was a key factor associated with individual MDA participation [24, 34, 47–50]. Respondents were more likely to participate in MDA campaigns if they had knowledge of the cause of malaria and that asymptomatic cases exist [47]. This indicates similar results to another mixed-methods study that found that the desire to volunteer in MDA was positively associated with higher likelihood of accepting the idea of asymptomatic malaria [50].

#### Intervention knowledge

Awareness and knowledge of the intervention itself was positively associated with MDA participation [47]. MDA participation was more likely to occur if villagers were familiar with the risks and benefits of MDA [49, 51], and the rationale for MDA [47–50]. More participants completed an entire MDA course if they received education on MDA from local health teams [34]. Conversely, villagers were less likely to participate if they had a poor understanding of or reported not being informed about MDA [24].

#### Attitudes and perceptions

MDA participation was associated with a concern about malaria [51, 52], a desire to improve one’s health [50], and a general confidence in the intervention [41, 48]. On the other hand, fear of certain MDA programme elements, such as blood tests, was strongly emphasized as reasons for non-participation [23–25, 47]. Additional concerns around real and perceived adverse side effects at times led to rumours that were linked to decreased participation [23, 25, 41].

#### Social dynamics

Social dynamics and interdependence within communities were related to individual MDA participation [41, 47, 50, 53]. Individuals often made decisions to participate in MDA on a household or group basis [41, 51, 53], where household heads had a strong influence on the participation of other household members [41]. MDA participation and refusals were also impacted by the degree of community cohesiveness and a tendency for social conformity [24, 28, 41]. Poor participation in one community was attributed to perceived affiliations of a MDA project with an opposing political group [50]. Wealthier community members and recent immigrants to a village did not consider themselves to be part of the wider community. These factors were related to a diminished sense of responsibility to participate in MDA campaigns [41, 46] and a low proportion of participation [46].

#### Personal characteristics

MDA participation was positively associated with older age, seeking malaria treatment at a government health centre for fever, literacy, and religion, as well as residency in certain villages [24, 34]. Mixed associations were found between certain ethnic backgrounds and willingness to participate [34, 47]. Occupation type and whether respondents had children were not significant factors associated with MDA participation [34].

## Discussion

This systematic review identified thirty publications from academic and grey literature sources focusing on factors influencing the uptake of two key malaria interventions, ITNs and MDA, in countries belonging to the ASEAN [23]. Several broad observations were noted among all included studies. First, no research was identified in Singapore, Brunei, Indonesia, or the Philippines. While the search was performed for all ASEAN countries, not all countries had evidence pertaining to them in the final publications that were identified in the search. This lack of research evidence may reflect the success of Brunei and Singapore, as well as several regions in Indonesia and the Philippines, where malaria-free status was already achieved in the 1980s [54, 55]. Programme managers in these countries should assess the findings of this review to determine its relevance to their current malaria monitoring efforts. Secondly, most (60%) publications identified in this review examined factors contributing to use of ITNs, with relatively limited evidence on the reasons for variation in individual MDA participation in Southeast Asian countries. This finding may be explained by the more recent implementation of MDA for malaria elimination in the region and highlights the need for continued implementation research in this area.

In addition to the included qualitative studies (n = 16), quantitative studies (n = 8) highlighted several predictors of intervention uptake, such as area of geographic residence. Reasons for ITN use and MDA participation across different geographic and sociodemographic groups were not widely explored in qualitative studies. Future studies may consider the use of mixed methodology to identify sub-groups, who may be more or less likely to uptake interventions for malaria prevention and their underlying reasons for non-intervention use.

Two out of the six themes influencing ITN use were emphasized above the others in the included publications. These were knowledge related to malaria and ITNs, as well as attitudes and perceptions towards ITNs. Attitudes generally towards malaria intervention were also strongly associated with likelihood of uptake [26, 40]. These findings align with evidence from countries outside Southeast Asia that indicates that knowledge and attitudes are key factors elsewhere as well [56]. Non-ITN use was related to inadequate programme focus on user attitudes regarding convenience for example, particularly among those belonging to mobile populations, including military personnel, migrant workers, and forest goers [26, 40]. These results emphasize the need for a clearer understanding of net design preferences among different population subgroups, for instance, to inform optimal net distribution campaigns. To help ensure inclusive protection of all sub-groups vulnerable to malaria infection, implementation campaigns must also align timing of ITN distribution campaigns with regional migratory patterns and implementation at work sites, including agriculture fields, fishing areas, or other places where large numbers of people congregate for work [37, 57].

In terms of MDA, the degree of knowledge about malaria and the intervention, as well as perceptions towards MDA were key factors influencing participation. These findings underscore the need for education and communication initiatives to complement delivery of integrated malaria programmes that include MDA, especially in endemic communities where knowledge about malaria and newer interventions are often lacking [37]. Alongside direct cost considerations, studies also emphasized indirect costs and benefits (i.e. free medical care). Future research, modelling of cost-effectiveness, and the design of MDA activities should include the role and nature of appropriate incentives as additional considerations. Finally, factors related to the theme social dynamics also uniquely described individual MDA participation. It is possible that trust plays a stronger role in shaping decisions to participate in MDA given its limited deployment in ASEAN countries compared to ITNs which have been in the context for a longer period of time [50, 58]. There is a need for continued formative research and strong collaboration between the scientific community and other stakeholders to coordinate malaria elimination strategies that are adapted to the local social context. These findings highlight the critical role of community engagement and engaging with local leaders to ensure interventions are not perceived negatively, to guide the implementation of MDA programmes especially [48, 53, 59].

### Strengths and limitations

This systematic review of two types of malaria intervention provides a timely overview of evidence from research and grey literature sources to assist in policymaking for Southeast Asian countries approaching malaria elimination. It provides the basis for developing strategies for maximizing adherence to drug regimens that the WHO identified as a key question in its 2019 Evidence Review Group Meeting report [60]. This review highlights potential research gaps about user preferences related to net design for further qualitative investigation. There are a number of limitations to this study that should be noted. First, it is possible that there may be some data duplication since some of the publications, particularly about MDA, may be from the same original malaria intervention project. Other potentially relevant publications may have also been excluded because they were not available in full text in the English language. Additionally, there was a lack of evidence about intervention uptake in a number of the ASEAN countries that ideally would have been included in this review. Given the types of study designs used in many of the included studies, it is likely that the results do contain some level of bias and thus appropriate caution should be taken when interpreting the results. Also, while there are summarized factors associated with intervention uptake across these various studies and contents, it is possible that differences in uptake may be related to variations in the receiving populations or implementing agencies that were not measured, compared or reported and thus could not be considered here.

## Conclusion

This systematic review described factors for ITN use and MDA participation among populations in the ASEAN region. Results of this study can support decision-making for policy-makers and programme managers involved in malaria control in Southeast Asia. Malarial disease and intervention knowledge and positive attitude towards interventions remain key factors associated with higher rates of uptake and participation in both of these forms of malaria intervention. There is a continued role for health educators in malaria prevention in Southeast Asia. There is also a need to maintain a focus on sociocultural and gender norms as well as details of local contexts of intervention, such as seasonal movement of subpopulations, to ensure successful implementation of malaria prevention strategies. Ongoing operational or formative research could lead to improvements in rates of ITN use and increasing levels of acceptability of MDA in remote and hard-to-reach mobile communities especially. In addition to ITNs and MDA, future reviews could examine the effectiveness and uptake of other kinds of malaria interventions such as larval source management and housing improvements. This review identified 30 unique studies looking at factors affecting ITN use or MDA participation in ASEAN nations. As countries move forward with plans to eliminate malaria by 2030, it is essential that intervention studies remain part of, and are drawn on to inform, these efforts.

## Supplementary Information

**Additional file 1. **Search Strategy. Detailed search string used for scientific database searching.

**Additional file 2. **Summary of publications discussing insecticide-treated bed net (ITN) use (n = 18). Summary of publications included in this review discussing insecticide-treated bed net (ITN) use.

**Additional file 3. **Summary of publications discussing mass drug administration (MDA) (n = 12). Summary of publications included in this review discussing mass drug administration (MDA).

**Additional file 4. **Themes describing factors related to ITN use. Themes describing factors related to ITN use.

**Additional file 5. **Themes describing factors related to MDA participation. Themes describing factors related to MDA participation.

## Data Availability

The datasets supporting the conclusions of this article are included within the article and its additional files.

## Abbreviations

ASEAN: Association of Southeast Asian Nations
ITN: Insecticide-treated bed net
LLIN: Long-lasting insecticidal net
MDA: Mass Drug Administration
PRISMA: Preferred Reporting of Items for Systematic Reviews and Meta-Analyses
WHO: World Health Organization
