# Supplementary material for: A systematic review of factors influencing participation in two types of malaria prevention intervention in Southeast Asia
Source: Malar J. 2021 Apr 20;20:195. doi: 10.1186/s12936-021-03733-y (PMC8056550; doi:10.1186/s12936-021-03733-y)
Supplement: Supplementary file 1 — Additional file 1. Search Strategy. Detailed search string used for scientific database searching. [file 12936_2021_3733_MOESM1_ESM.pdf]

Additional File 1

Supplementary Table: Scoping Review Search Terms

#	Searches	Notes
1	malaria/ or plasmodium vivax malaria/ or malaria falciparum/	
2	malaria control/	
3	malaria control.mp.	
4	Plasmodium falciparum/	
5	plasmodium falciparum.mp.	
6	malaria.mp. [mp=title, abstract, heading word, drug trade name, original title, device manufacturer, drug manufacturer, device trade name, keyword, floating subheading word, candidate term word]	Search terms for malaria
7	paludism*.mp. [mp=title, abstract, heading word, drug trade name, original title, device manufacturer, drug manufacturer, device trade name, keyword, floating subheading word, candidate term word]	
8	bed net/ or insecticide treated net/	
9	bed net*.mp. [mp=title, abstract, heading word, drug trade name, original title, device manufacturer, drug manufacturer, device trade name, keyword, floating subheading word, candidate term word]	
10	bednet*.mp. [mp=title, abstract, heading word, drug trade name, original title, device manufacturer, drug manufacturer, device trade name, keyword, floating subheading word, candidate term word]	
11	mosquito net*.mp. [mp=title, abstract, heading word, drug trade name, original title, device manufacturer, drug manufacturer, device trade name, keyword, floating subheading word, candidate term word]	Search terms for ITNs
12	treated net*.mp. [mp=title, abstract, heading word, drug trade name, original title, device manufacturer, drug manufacturer, device trade name, keyword, floating subheading word, candidate term word]	
13	(interceptor and (bednet* or net*)).mp. [mp=title, abstract, heading word, drug trade name, original title, device manufacturer, drug manufacturer, device trade name, keyword, floating subheading word, candidate term word]	
14	8 or 9 or 10 or 11 or 12 or 13	Bed net concept
15	mass drug administration/	
16	mass drug administration.mp.	
17	exp antimalarial agent/	
18	antimalaria*.mp. [mp=title, abstract, heading word, drug trade name, original title, device manufacturer, drug manufacturer, device trade name, keyword, floating subheading word, candidate term word]	Search terms for mass drug administration
19	anti-malaria*.mp. [mp=title, abstract, heading word, drug trade name, original title, device manufacturer, drug manufacturer, device trade name, keyword, floating subheading word, candidate term word]	
20	infection prevention/	
21	drug therapy/ or chemoprophylaxis/	
22	exp southeast asian/	
23	southeast asian.mp.	
24	exp Southeast Asia/	
25	south east asia.mp.	
26	Viet Nam/	
27	Viet Nam.mp.	
28	Cambodia/	
29	Cambodia.mp.	
30	Myanmar/	
31	Myanmar.mp.	
32	Burma.mp.	
35	Laos/	
36	Laos.mp.	Search terms for Southeast Asia
37	Thailand/	
38	Thailand.mp.	
39	Brunei Darussalam/	
40	Brunei Darussalam.mp.	
41	Indonesia/	
42	Indonesia.mp.	
45	Timor-Leste/	
46	Timor-Leste.mp.	
47	Malaysia/	
48	Malaysia.mp.	
49	Greater Mekong*.mp.	

50	22 or 23 or 24 or 25 or 26 or 27 or 28 or 29 or 30 or 31 or 32 or 33 or 34 or 35 or 36 or 37 or 38 or 39 or 40 or 41 or 42 or 43 or 44 or 45 or 46 or 47 or 48 or 49	Country concept
51	1 or 2 or 3 or 4 or 5 or 6 or 7	Malaria concept
52	15 or 16 or 17 or 18 or 19 or 20	Drug concept
53	14 and 50 and 51	Full net concept
54	50 and 51 and 52	Full drug concept
