# Supplementary material for: A systematic review of factors influencing participation in two types of malaria prevention intervention in Southeast Asia
Source: Malar J. 2021 Apr 20;20:195. doi: 10.1186/s12936-021-03733-y (PMC8056550; doi:10.1186/s12936-021-03733-y)
Supplement: Supplementary file 2 — Additional file 2. Summary of publications discussing insecticide-treated bed net (ITN) use (n = 18). Summary of publications included in this review discussing insecticide-treated bed net (ITN) use. [file 12936_2021_3733_MOESM2_ESM.docx]

**Additional file 2:**  Summary of publications discussing insecticide-treated bed net (ITN) use (n=18).

| **Reference** | **Location** | **Population** | **Design** | **Statistical analysis** | **Publication aims** | **Summary** |
| --- | --- | --- | --- | --- | --- | --- |
| Aung et al., 2016 | Myanmar | 4679 household heads | Cross-sectional | Descriptive statistics, bivariate, multivariate regression | Describe effective ITN ownership and use among all household members and children under five years in eastern Myanmar. | Low ITN ownership and use overall, but higher rates of ownership among groups vulnerable to malaria. Malaria knowledge strongly linked to effective ITN use. |
| Gryseels et al.,  2019 | Cambodia | 151 community members | Qualitative | NA | Adapt and expand the concept of appropriateness of bed nets in a malaria control and elimination intervention context. | Long-lasting insecticide-treated bed nets (LLINs) were not always appropriate to their sociocultural context, which influenced variability in use. |
| Gryseels et al., 2015 | Cambodia | 300 youth aged 13-17, 246 household leaders | Qualitative | Proportions and tabulations, descriptive statistics | Aims to understand sociocultural factors relate to malaria infection among the Jarai ethnic population living on both sides of the Cambodia-Vietnam border. | Jarai youth have low protection from malaria by bed nets due to age-specific sleeping patterns and structures, low bed net use, and cross-border mobility. |
| Kitidamrongsuk et al., 2016 | Thailand | 1674 household members | Cross-sectional | Descriptive statistics, multivariate analysis | Identify determinants of impregnated net ownership and use in rural Thai residents. | Net size is a major obstacle of net use among larger households. Irregular use of electric fans at night was positively associated with sleeping under a bed net, as well as ethnicity, insecticide-residual spray access. |
| Kyi Kyi Win Oo, 2012 | Myanmar | General population affected by Cyclone Nargis | Thesis, Grey literature | NA | Investigate the main reasons for using or not using of Business Kind Myanmar (BKM) bed nets in the household. | Reasons for use varied based on season, livelihood patterns, and household sleeping arrangements. Some nets purchased only for summer use while heavier nets used for colder seasons. |
| Leake et al., 2019 | Malaysia | 711 adult household members | Mixed methods | Descriptive statistics | Identify factors influencing use of insecticide impregnated bednets to control malaria. | Bed net use rates differed between villages, which was attributed mainly to television viewing. Television viewing and alcohol drinking may reduce motivation for bed net use. |
| Linn et al., 2015 | Myanmar | 3230 adults from high-risk migrant communities in the Regional Artemisinin Resistance Initiative (RAI) | Mixed methods | Descriptive statistics, bivariate and multivariate analysis | Assess the ownership and utilization of ITNs and to understand the barriers to distribution and utilization of ITN | Household sizes with fewer than six people had higher odds of having sufficient bed nets. Reasons for non-use include insufficient or lack of availability of ITNs, inconvenience of carrying nets to work sites, and dislike and discomfort due to heat, smell, and health concerns related to insecticide. Transportation to reach remote communities was a major barrier for ITN distribution. |
| Liu et al., 2015 | Myanmar | 3351 household members | Mixed methods | Bivariate analysis | Identify coverage of both treated (ITNs and LLINs) and untreated bed nets, and also to determine which factors influence bed net use and maintenance. | Bed net use was positively associated with factors related to knowledge (i.e. household knowledge of malaria transmission and the role of ITNs in malaria prevention), income (e.g. belonging to a low family wealth index and a family with a primary income source from agricultural work ,and residence (i.e. living in lowland and foothills in households with thatched roofing). |
| Min et al., 2020 | Myanmar | 4597 children under five | Cross-sectional | Bivariate and multivariate analysis | To examine use of nets and associated factors, care seeking behaviour among caregivers and factors, uptake of malaria testing among those with fever in the last two weeks. | Non-use of bed nets was associated with residing in malaria elimination, urban, and delta or hilly regions, and belonging to households in the highest wealth quintile. |
| Nofal et al., 2019 | Cambodia, Vietnam, Myanmar, Thailand | 22 studies | Systematic review | NA | Provide an overview of the qualitative research on behaviours and perceptions that influence uptake of and adherence to malaria interventions among forest-goers in the Greater Mekong Subregion. | Forest-goers do not use nets related to hot environment of forests, inconvenience of carrying to work sites, and insufficient access to nets. Some studies cited lack of affordability, preferences for non-treated nets, and perceived safety and effectiveness of nets. Use of nets was also influenced by household size, where larger nets were preferred for larger families when not working in forests. |
| Nyunt et al., 2014 | Myanmar | 345 migrant workers in palm oil and rubber plantation sites | Mixed methods | Descriptive statistics | To explore knowledge, distribution and utilization of ITNs. | The major reason for non-use of ITNs was working over night at work sites and the major reasons for use of ITNs were free distribution of nets and preventing mosquito bites. Perceived risk of malaria was higher among consistent ITN users compared to nonusers. Qualitative findings suggest that perceived poor net durability, discomfort, and misuse are key ITN challenges. |
| San Oo et al., 2013 | Myanmar | 256 household heads > 18 y | Cross-sectional | Proportions and tabulations, descriptive statistics | To assess knowledge of malaria, usefulness of bed net, perception on uses of nets, observe ownership and identify treatment seeking behaviour for malaria. | Low knowledge levels on malaria and ITN use, as well as poor attitude is a need for increasing availability of information on ITNs. |
| Sri-aroon et al., 1998 | Myanmar | 184 mothers and caretakers with children < 11y | Mixed methods | Descriptive statistics, bivariate analysis | To examine the effect of socio-behavioural backgrounds of mothers on decision making in terms of bed nets. | Knowledge of malaria prevention and information gained about use of impregnated bednets among mothers and caregivers were linked to the use of impregnated bed nets.  Knowledge of disease causes, transmission, severity, and susceptibility to malaria were not found to be significantly associated with net use. |
| Stewart et al., 2003 | Multi-country | General population | Report, Grey literature | NA | Discuss a range of factors influencing usage of ITNs. | Economic, environmental, entomological, and social factors affecting net use were identified, with social factors standing out as the dominant reason affecting net use. |
| Tun, K and Soe, M., 2009 | Myanmar | Household heads or adults > 18y | Quantitative | Descriptive statistics | To assess knowledge, attitude and practice of community on ITN. | The top reason for not using ITNs was not knowing about ITNs and lack of ITNs. Other reasons mentioned to lesser degree include not being able to afford purchasing ITNs and disliking its use, not knowing where to buy ITN treatment tablets. |
| United States Agency for International Development (USAID), 2012 | Myanmar, Thailand, Cambodia | General population | Report, Grey literature | NA | Provide a review of malaria prevention strategies for countries in the Greater Mekong Subregion. | Overview suggests that consumer preferences and poor affordability affects ITN use. Nets used for nuisance biting, malaria prevention, and privacy. |
| Vilay et al., 2019 | Laos | 313 military personnel | Mixed methods | Descriptive statistics | This study determined the prevalence of malaria infection and assessed knowledge, perception, and preventive and treatment behavior regarding malaria among military personnel in two southern provinces in Lao PDR. | Participants viewed nets as important and necessary, but these measures were not always available. Despite being distributed for free, in some cases, participants had to purchase their own tools at markets. Data suggests that nets were not used due to a lack of sufficient supply of ITNs, inappropriate location for hanging nets, and security inspections. |
| Wharton-Smith, A and Shafique, M., 2014 | Myanmar | General population | Report, Grey literature | NA | Outlines findings of a behavioral study that was done to assess consumer preferences and barriers to use of LLINs in Myanmar. | Lack of knowledge of malaria was a common reason cited for non-use of nets. Net preferences such as texture and perceived adverse effects affected use of LLINs. |
| Welch & Fuster, 2012 | Cambodia | 168,232 household members | Cross-sectional | Descriptive statistics, bivariate, and multivariate analysis | Assess current disparities in access to ITNs, what factors may be associated with disparities in access  and the progress of antimalaria interventions. | Problematic distance from ITN distributors, rural location, and poverty were all associated with greater unlikelihood of possession of least one household ITN. |
