# Supplementary material for: A systematic review of factors influencing participation in two types of malaria prevention intervention in Southeast Asia
Source: Malar J. 2021 Apr 20;20:195. doi: 10.1186/s12936-021-03733-y (PMC8056550; doi:10.1186/s12936-021-03733-y)
Supplement: Supplementary file 3 — Additional file 3. Summary of publications discussing mass drug administration (MDA) (n = 12). Summary of publications included in this review discussing mass drug administration (MDA). [file 12936_2021_3733_MOESM3_ESM.docx]

Additional file 3: Summary of publications discussing mass drug administration (MDA) (n=12).

| **Reference** | **Location** | **Population** | **Design** | **Statistical analysis** | **Objective** | **Summary** |
| --- | --- | --- | --- | --- | --- | --- |
| Adhikari et al. (2018) | Laos | 281 adult household heads | Cross-sectional | Descriptive statistics, bivariate and multivariate analysis | How community engagement and sociocultural context affected uptake of MDA. | Awareness of asymptomatic malaria was positively associated with knowledge of MDA rationale and desire to participate in future MDA campaigns. |
| Adhikari et al. (2018) | Laos | 100 community members | Qualitative | Multivariate analysis | Reasons for participation in targeted malaria elimination. | Complete participation in Targeted Malaria Elimination (TME) was significantly associated with participation in community engagement activities, knowledge that the blood tests were for malaria, family members’ participation at TME, and perceptions that TME was worthwhile. |
| Kaehler et al. (2019) | Thailand, Myanmar, Cambodia, Laos, Vietnam | 32 policymakers, principal investigators | Qualitative | NA | Identify attitudes towards and perceptions of MDA for malaria elimination among policymakers and malaria experts. | Four challenges were identified that influenced uptake of MDA in Southeast Asia, including weak evidence base for MDA, determining appropriate implementation sites, national and local politics, and implementation issues that affected eventual participation rates. Community engagement was an important facilitator of MDA participation. Rumors was a major barrier that had significant negative impacts on population coverage, as well as historical trauma from past MDA projects and difficulty in understanding MDA rationale. |
| Kajeechiwa et al. (2016) | Myanmar | 848 household members | Mixed methods | Descriptive statistics, multivariate analysis | Identify reasons for participation or non-participation in drug administration campaign. | People who felt they received sufficient information about the campaign were more likely to participate in MDA. Respondents from certain villages (KNH, TPN) were more likely to participate in MDA from other villages (HKT, TOT). Refusals in HKT was associated with Burman ethnicity, who were mostly store owners that had to close shops to participate in MDA. A universal reason for non-participation was inadequate understanding of malaria intervention. Those who didn’t know causes of malaria were more likely to not participate in MDA and villagers who recognized malaria symptoms ie. headache,were more likely to participate. Other reasons include absences from village at time of campaign and unconfirmed rumours about adverse effects. |
| Kajeechiwa et al. (2017) | Myanmar | 848 household members | Qualitative | NA | Describes the community engagement  efforts for a pilot malaria elimination project, the challenges encountered and  lessons learnt. | Broad factors impacting implementing include difficult in reaching certain villages was difficult due to rainy season and logistical issues, as well as seasonality of implementation that coincided with working schedules of villagers. Challenges in explaining difficult concepts of malaria, as well as rumors underpinned by fear of adverse events, contributed to hesitation to participate. |
| Nguyen et al. (2017) | Vietnam | 148 villagers | Cross-sectional | Multivariate analysis | To explore why some people participate in  MDAs and others do not. | A critical difference between participation and non-participation was being adequately informed about the campaign, as the more informed one was about MDA’s purpose, the more likely they were to participate and complete entire course. There was a strong association between participation and sensitization by an informant. Demographic variables positively associated with net use include village residency, older age, ethnicity, literacy, and religion. Non-significant participation factors included occupation and whether they had children. |
| Nofal et al. (2019) | Cambodia, Vietnam, Myanmar, Thailand | 22 studies | Systematic review | NA | Provide an overview of the qualitative research on behaviours and perceptions that influence uptake of and adherence to malaria interventions among forest-goers in the Greater Mekong Subregion. | Participation was affected by concerns related to the safety of blood tests and perceived side-effects of antimalarial drugs or fears that test results would reveal illegal forest work. Non-participation linked to being absent from village during time of MDA implementation. |
| Pell et al. (2019) | Myanmar, Vietnam, Cambodia, Laos | 1591 community members, leaders, and trial staff | Mixed methods | Descriptive statistics, multivariate analysis | Examines the impact  of the community engagement, local social context and study design on MDA coverage. | 100% completion of all three rounds of MDA was positively associated with having heard of MDA and malaria-related knowledge. Villagers’ had a general appreciation for value of addressing malaria and improved intervention confidence through provision of essential health services.  The role of trust was highlighted as a determinant and barrier, demonstrated by the negative impact of rumors and community divisions on participation and hesitation due to fear of adverse health events, blood tests, and historical trauma. Coverage didn’t decrease in absence of cash compensation. |
| Pell et al. (2017) | Cambodia | 30 adult household members | Qualitative | NA | Explores the factors that influenced mass anti-malarial administration  coverage within a clinical trial. | Individual level factors contributing to participation in MDA included familiarity and concern about malaria and aim of malaria elimination, awareness of MDA. Contextual factors include community dynamics, local politics, the tendency for village conformity, and group decision making. Non-participation primarily related to fear of adverse events and blood drawing. Community engagement addressed community health concerns and its effects mediated by trust relationships with study staff. Coverage not decreased in absence of cash compensation for participation. |
| Peto et al. (2018) | Cambodia | 188 villagers | Mixed methods | NA | Discusses lessons learned about community responses to two mass anti-malarial administrations. | MDA coverage did not change in the absence of financial incentives, suggesting that its role in participation may be overstated. Tendency for conformity were linked to high coverage, while lower coverage was seen in communities with stronger political and social divisions. Awareness of asymptomatic infection was also associated with higher participation. Villagers’ appreciated increased medical attention and convenience of house-to-house delivery. |
| Sahan et al. (2017) | Myanmar | 45 villagers and study staff | Qualitative | NA | How these factors influenced attitudes and behaviours  towards Targeted Malaria Treatment (TMT) | Trust had a key influence on participation in a setting with limited health infrastructure. Trust was founded not only on the immediate short-term provision of additional healthcare, but also through demonstrating a commitment to the local health needs. Decisions about participation were also made in the background of malaria-related knowledge and at times, were made on behalf of others. |
| Shoklo Malaria Research Unit | Myanmar | General population | Report, Grey literature | NA | Discusses results from a mass drug administration campaign, including reasons for non-participation. | MDA was overall well-accepted. Non-participation was due to refusals, ineligibility, and incomplete participation. Among those who refused participation, the main reason was not wanting to take medicine due to not feeling sick, not trusting western medicine, or not perceiving malaria as a problem. Participants who had other conditions also reported not wanting to take another drug. Community engagement was key for community participation as it fostered trust. Rumors in some villages had a significant negative impact on participation rates that recovered after rumor control from staff. |
