# Supplementary material for: A systematic review of factors influencing participation in two types of malaria prevention intervention in Southeast Asia
Source: Malar J. 2021 Apr 20;20:195. doi: 10.1186/s12936-021-03733-y (PMC8056550; doi:10.1186/s12936-021-03733-y)
Supplement: Supplementary file 4 — Additional file 4. Themes describing factors related to ITN use. Themes describing factors related to ITN use. [file 12936_2021_3733_MOESM4_ESM.docx]

Additional file 4: Themes describing factors related to ITN use.

| **Themes** | **Papers** |
| --- | --- |
| Access and delivery | Transportation was a major barrier for distribution due to village remoteness; restrictive procurement policies delayed; Respondents used ITN if they were available and in sufficient supply. |
| Cost and benefits | The cost of ITNs was an important factor for poor families who could not afford to replace nets once damaged and families often used bed nets for purposes other than malaria prevention such as for warmth. |
| Malaria knowledge | Use among households and children under five were significantly associated with household members and mothers that understood malaria transmission. One study found no significant associations with net use and knowledge of causes and transmission. |
| Intervention knowledge | Use among households and children under five were significantly associated with understanding the role and benefits of bed nets in prevention. |
| Attitudes and perceptions | Perceived safety and comfort influenced net use. Key challenges for use related to excessive heat, unpleasant smells, and general discomfort due to insecticide treatment. Past experiences of malaria prompted use of bed nets as well. Low durability after washing led to perception of low effectiveness. Only one study did not find significant association between use and attitudes towards bed nets. Preferences for larger market nets with smaller mesh that can accommodate larger households and also afford more privacy. Net use not prioritized after late-night TV watching and participation in social events. Inconvenient to carrying nets to work site. |
| Personal characteristics | Net use was associated with residence, children under five in rural households vs. urban households, those who did not have problematic distance to a health facility, and households living in lowlands and foothills vs. mid and upper hills due to more access to commercial bed nets. Significant association between having sufficient bed net use and having a household size <6. Non-possession of at least 1 ITN in the household was associated with age group and distance to health facility. Less likelihood of use among household members and children under five was associated with a household headed by a skilled or businessman compared to those headed by a farmer or fisherman. Net use and likelihood of a household owning at least 1 net was associated with having a high family wealth index. Being in the richest quintile was also associated with net use. |
