# Supplementary material for: A systematic review of factors influencing participation in two types of malaria prevention intervention in Southeast Asia
Source: Malar J. 2021 Apr 20;20:195. doi: 10.1186/s12936-021-03733-y (PMC8056550; doi:10.1186/s12936-021-03733-y)
Supplement: Supplementary file 5 — Additional file 5. Themes describing factors related to MDA participation. Themes describing factors related to MDA participation. [file 12936_2021_3733_MOESM5_ESM.docx]

Additional file 5: Themes describing factors related to individual MDA participation

| **Themes** | **Description** |
| --- | --- |
| Access and Delivery | Isolated settlements made travel difficult and led to high staff turnover that delayed relationship building process. Participation linked to slow but critical government approval of MDA studies. In one study, MDA participation was significantly associated with seeking malaria treatment at a government health centre for fever. No participation due to village absence at time of campaign. Participation facilitated by community engagement (CE) and collaboration with village volunteers, in part because it responded to AE concerns. |
| Costs and incentives | Direct and indirect costs were related to participation in MDA. Community members also strongly valued additional incentives including the provision of free essential medical services, installation of water pumps, attention to individual health concerns during door-to-door MDA delivery. There is mixed evidence around the effect of financial compensation. |
| Malaria knowledge | Participation related to knowledge of asymptomatic malaria, and knowing that malaria can be diagnosed through blood tests. Respondents participated if they felt they received sufficient information on and also recommend programme to nonparticipants. Participation was unsuccessful due to difficulties in explaining concept of asymptomatic malaria and was less likely if respondents didn’t know causes of malaria. |
| Intervention knowledge | Universal reason for non-participation was poor understanding of intervention rationale, as well as awareness of MDA and benefits. Community engagement activities (i.e. project meetings) promoted concept and rationale of MDA. |
| Attitudes and perceptions | **Positive attitudes:** Participation was underpinned by the perception that TME was important and liking all aspects of TME, being concerned about malaria, and awareness of MDA. Recognizing malaria as an important health hazard was another reason for participation. Appreciation for 1-on-1 attention to suffering and convenient ability to ask staff questions during house to house MDA administration. Showing caring and providing essential health assistance boosted confidence in intervention. Financial incentives in subsequent rounds encouraged participation but its absence did not influence participation and not cited as a major reason for participation. Participation continued despite side effects, which was motivated by concerns about suffering from malaria. Participation was highly motivated by ancillary healthcare, and highly valued.  **Negative attitudes:** Negative perceptions involved fear of drug safety, adverse events, and blood tests, where results were believed to have potential to identify illegal forest work Lower participation rates were observed in subsequent rounds following these health complaints, concerns related to historical trauma from past MDA trials and spread of unconfirmed rumours. Drop in coverage in round 2 due to attitude changes in trust. |
| Social dynamics | Participation was more likely if all members from a household participated. Decisions to participate were influenced heavily by degree of community cohesiveness, where lower levels of coverage were caused by strong political divisions or a lack of sense of belonging among migrants new to the community. Decisions to participate or refusals also occurred in clusters. |
| Personal characteristics | Religion, literacy, and ethnicity was significantly associated with MDA participation, although ethnicity did not seem to affect willingness to participate in another qualitative study. |
